# Utilization of Pineapple Fruit Waste in Greener Alternative Agents for Thai Silk Pretreatment and Acid Dyeing Wastewater Treatment

**DOI:** 10.3390/ma18030674

**Published:** 2025-02-03

**Authors:** Jantip Setthayanond, Patintida Chuenjai, Piyaporn Kampeerapappun, Porntip Tooptompong

**Affiliations:** 1 Department of Textile Science, Faculty of Agro-Industry, Kasetsart University, Bangkok 10900, Thailand; jantip.s@ku.ac.th (J.S.); ppatintida@gmail.com (P.C.); 2 Faculty of Textile Industries, Rajamangala University of Technology Krungthep, Bangkok 10120, Thailand; piyaporn.k@mail.rmutk.ac.th

**Keywords:** Thai silk, pineapple fruits, degumming, adsorbent materials, sustainable use

## Abstract

Pineapple, extensively cultivated in tropical and subtropical regions, contains bromelain, a protein-digesting enzyme that is highly valued in the food and beverage industries. Pineapple residues from food processing retain these enzymes and can be repurposed for silk processing. This research utilized Smooth Cayenne pineapple juice as a degumming agent and its pulp as an adsorbent for dyeing effluent treatment. Pineapple juice, containing bromelain with a protease activity of 16.40 µg/mL·min, effectively removed 22% of sericin from raw silk using a liquid ratio of 30:1 at pH 7 and 60 °C for 60 min. Unlike alkaline degumming, which weakened silk fibers (maximum load 6.18 ± 1.56 N), pineapple juice-treated silk retained higher strength (maximum load 7.80 ± 1.32 N), offering a gentler alternative. The remaining pineapple pulp, after juice extraction, was processed into a porous adsorbent with a surface area of 3.63 m^2^/g and a pore size of 6.15 nm. This material absorbed acid dyes effectively at pH 5, the normal pH used in the acid dyeing of silk. Valorizing pineapple residues reduces chemical use, energy consumption, and environmental impact while lowering production costs and enhancing local resources.

## 1. Introduction

Pineapple (*Ananas comosus* (L.) Merr.), a member of the Bromeliaceae family, is among the most valuable tropical fruits globally. Its primary production is concentrated in South and Central America and Southeast Asia, regions characterized by favorable climates and advanced agricultural practices that facilitate large-scale cultivation [1]. Pineapple is a wonderful source of vitamins and minerals, offering numerous health benefits [2,3]. Renowned for its high vitamin C content, it strengthens the immune system, promotes skin health, and functions as a powerful antioxidant [2,3,4,5,6]. Its significant manganese levels contribute to bone strength and energy metabolism, while potassium supports blood pressure regulation and optimal muscle function [7,8].

Thailand ranks as the fourth-largest producer and exporter of pineapples in the world, showcasing its prominent position in the global pineapple industry [9]. Thailand is home to 75 pineapple processing companies, collectively generating approximately 200 tons of waste daily [10]. This waste not only arises from freshly consumed fruits, commercial juices, and canned or frozen goods, but also from harvesting and processing activities [11].

The utilization of waste is a sustainable strategy for waste management, emphasizing resource optimization and waste reduction. Pineapple waste, in particular, has been extensively studied for various applications. Choonut et al. [12] extracted bioethanol from leftover pineapple peel with a yield of 9.69 g/L, while Casabar et al. [13] extracted 5.98 g/L from pineapple fruit peel after 48 h of fermentation. Kumar et al. [14] utilized pineapple crown waste fiber to develop high-strength paper. Pineapple waste has also been used for environmental and industrial purposes, such as removing toxic contaminants [15]. It serves as lignocellulosic feedstock for polyhydroxybutyrate (PHB) production [9,16], creates decomposable pots [9], and enables the green degumming of silk [17]. Additionally, pineapple leaves were studied for soda pulping to produce paper [18] and to manufacture high-quality blended yarn [19]. Even under-grade fresh pineapples, often considered waste, can be transformed into value-added products like dehydrated pineapple, jams, and baked goods. These diverse applications highlight the significant potential of pineapple waste in promoting sustainability.

**Figure 1:**
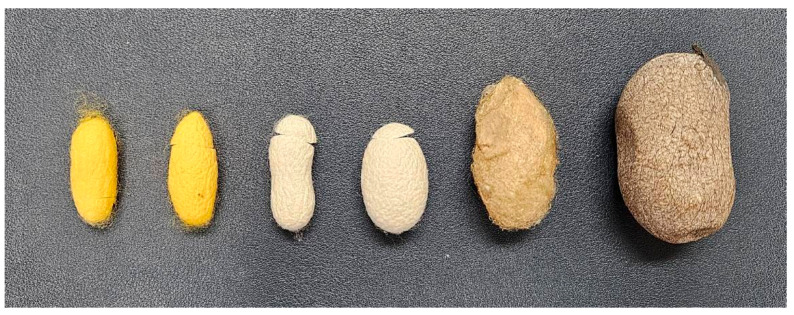
Various silk cocoon types (from left): Nang Noi Thai silk, Nang Lai Thai silk, UB1 Japanese silk, J108 Chinese silk, Indian Muga silk, and Indian Tassah silk.

Silk, a natural protein fiber regarded as the “queen of fibers”, is widely utilized in various applications such as textiles, biotechnology, composites, and the biomedical field [2,3]. Silkworms produce two primary fibers, known as fibroin filaments, which are coated with sericin, a sticky protein substance. Each type of silk cocoon has its own characteristics and sizes, as seen in Figure 1. Mulberry silk has smaller cocoons compared to wild silk, with a fiber length of approximately 1600 m [20]. Traditional Thai silk cocoons are yellow in color after reeling, rendering lustrous, yellow silk filaments [21]. Local Thai silk fabric is normally produced by communities of farmers during their off-farming season. It is a household production process that includes planting mulberry trees, cultivating silkworms, reeling silk, dyeing, and weaving fabric. Thai silk is well known for its elegance and luster, and is mostly produced by the communities located in the northern and northeastern area of Thailand. Each silk-producing community has unique silk products, and different production areas can claim an identity based on silk. A number of community enterprises for Thai silk production are then established to raise community earning and well-being, thereby contributing to the long-term economic sustainability of the country [22,23].

Prior to dyeing, sericin must be removed to enhance the handling properties, dyeability, and the softness of silk filaments [24,25,26]. This process, known as degumming, can be carried out using various methods such as alkaline degumming, acidic degumming, soap degumming, enzymatic degumming, high-temperature and high-pressure degumming, and CO_2_ supercritical fluid degumming, each of which has its own advantages and disadvantages [2,3,4,5,27,28,29]. However, traditional degumming methods, such as alkaline degumming, involve harsh chemical treatments that can have a negative impact on the environment. As a result, there is increasing interest in more sustainable, eco-friendly degumming methods, including enzyme degumming [30,31], CO_2_ supercritical fluid degumming [32,33], steaming degumming [34], and ultrasonic degumming [35]. Enzyme degumming, particularly that performed using protease enzymes, is the most commonly used method for degumming silk due to its environmentally friendly nature, making use of biodegradable substances and milder conditions that maintain silk quality and reduce environmental impact [36].

Bromelain, a mixture of protease enzymes, is primarily found in various parts of the pineapple plant in varying quantities, such as in the flesh, peel, core, stem, and crown [37]. Pineapple stem bromelain, known for its wide applications in the food, pharmaceutical, and health industries, is relatively expensive, whereas fruit bromelain can be easily extracted along with other existing compounds as a pineapple juice. However, it is not commercially viable [38]. In the countries with large-scale pineapple cultivation like Thailand, the acquisition of pineapple fruit bromelain is more simply and cheaply accomplished from pineapple fruit waste during harvesting and food processing. Additionally, the leftover pineapple pulp after juice extraction can likely be used to create adsorbent materials for dye removal from dyeing effluent.

**Figure 2:**
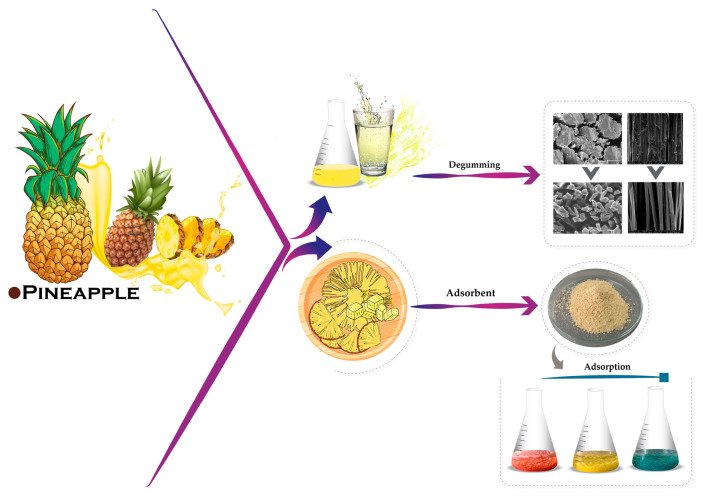
The utilization of pineapple fruit for silk pretreatment and dye effluent adsorption.

The remaining pineapple pulp after extracting the juice, as well as the pulp waste in pineapple processing factories, primarily consists of cellulose [39]. Pineapple pulp has the potential to be developed into adsorbent materials for treating dye effluent after the silk dyeing process. Acid dyes are synthetic dyes that are commonly used to dye silk, as they provide brilliant shades and good fastness properties for practical uses. However, when large amounts of acid dyes are released into water resources, this can cause serious environmental pollution [40,41,42,43,44,45]. As such, in this research, attention is paid to the use of pineapple by-products to raise the environmental friendliness of Thai silk processing, i.e., degumming and acid–dye effluent treatment. This involves using pineapple juice for silk sericin removal, while the pineapple pulp is studied as an adsorbent material for the development of dye effluent remedies after the silk acid dyeing process, aiming to achieve the complete utilization of pineapple fruit waste (Figure 2). A simple and feasible method could be available for community-based silk-producing enterprises, allowing them to accomplish greener silk production and raise the environmental value of the Thai silk processing and products. Upcycling pineapple fruit waste into dye adsorption material and silk degumming agents is a promising step towards sustainable practices in both the textile and waste management industries.

## 2. Materials and Methods

### 2.1. Materials

Smooth Cayenne (Pattavia) pineapple was purchased from Mahanark market, Thailand. Fine-filament, yellow Thai silk was obtained from Chaiyaphum province, Thailand. The adsorbent materials, derived from tamarind seed testa from our previous works [46,47] and activated carbon (model Carbon 1L) from Mozuma, were used in comparison with the pineapple pulp adsorbent. Three acid dyes, viz. C.I. Acid Orange 67, C.I. Acid Red 142, and C.I. Acid Blue 62, purchases from Phua Kiam Seen Co., Ltd. (Bangkok, Thailand), were chosen for the adsorption study. *L*-tyrosine, casein, and Bovine Serum Albumin (BSA) standard, obtained from Sigma-Aldrich (St. Louis, MO, USA), were used in protease activity analysis. C.I. Direct Red 80 (DyStar, Samutprakarn, Thailand) was employed for the sericin staining test. The other chemicals used were of a laboratory grade.

### 2.2. Methods

A 1 kg Smooth Cayenne (Pattavia) pineapple yielded approximately 700 mL of pineapple juice and left about 380 g of pulp after juice extraction. The pineapple juice, extracted by a cold-pressed juicer and filtered through T60 mesh screen fabric, was used to degum silk, while the pulp was used to prepare adsorbent material for residual acid dye treatment, as follows.

#### 2.2.1. Study of Silk Degumming with Pineapple Juice

The degumming of raw, fine yellow Thai silk [48] was conducted using pineapple juice with a liquor ratio (LR) of 30:1. The pH of the pineapple juice was brought to 7 before we degummed the silk at 60 °C. After degumming, the silk filaments were rinsed thoroughly with water and dried at room temperature. This process was compared to the conventional alkaline degumming method, using 2 g/L Na_2_CO_3_ along with 10 g/L wetting agent at a liquor ratio of 30:1. Alkaline degumming was conducted at 95 °C for 45 min as this is a commonly used silk degumming method [49,50,51]. Afterward, the silk was rinsed with warm water (about 60 °C). This was followed by rinsing in cold water (room temperature) rinsing before air drying.

The degummed silk was examined to establish degumming efficiency, the protein content in the solution, surface characteristics, water absorption, tensile strength, elongation, and the dye staining of sericin.

The degumming efficiency (E) was calculated based on the amount of sericin removed using the gravimetric method, as shown in Equation (1):

(1) $$E\;(\%)=\frac{(M_{0}-M_{1})\;}{M_{0}}\times 100$$

where *M_0_* and *M_1_* are the dry weight of silk before and after degumming, respectively [34,51].

#### 2.2.2. Study of Acid Dye Adsorption Efficiency of Pineapple Pulp

The pineapple pulp obtained after extracting pineapple juice was treated in boiling water to achieve the complete removal of sugar. It was then oven-dried and pulverized into a fine powder using a grinder at a speed of 12,000 rpm for 2 min. The adsorbent material powder prepared from pineapple pulp was used to study the adsorption efficiency of three acid dyes, viz. C.I. Acid Orange 67, C.I. Acid Red 142, and C.I. Acid Blue 62. The adsorption behavior of the adsorbent prepared from pineapple pulp was also investigated using Langmuir and Freundlich Isotherms. An adsorption study was conducted in a cylindrical container with a 5 cm diameter, using 1 g of adsorbent per 30 mL of the 50 mg/L dye solution.

The efficiency of acid dye adsorption (A) was analyzed from the absorbance values of the acid dye solutions before and after adsorption process using a UV-Vis spectrophotometer (Equation (2)). The wavelengths at maximum absorption of C.I. Acid Orange 67, C.I. Acid Red 142, and C.I. Acid Blue for this analysis were 400, 500, and 595 nm, respectively.

(2) $$A\;(\%)=\frac{(Abs_{0}-Abs_{1})\;}{Abs_{0}}\;\times \;100$$

where $Abs_{0}$ and $Abs_{1}$ are the absorbances of the dye solution before and after adsorption process, respectively.

The adsorption behaviors of the adsorbent material according to Langmuir (Equation (3)) and Freundlich Isotherms (Equation (4)) were examined as follows:

(3) $$\frac{C}{Q}=\frac{1}{q}C+\frac{1}{K_{L}q}$$

where Q is the amount of acid dye adsorbed per unit weight of the adsorbent, q is the maximum amount of acid dye adsorbed in a monolayer form, K_L_ is the Langmuir constant, and C is the concentration of adsorbed acid dye.

(4) $$\log\;Q=\left(\frac{1}{n}\;\right)\;logC+logK_{F}$$

where Q is the amount of acid dye adsorbed per unit weight of the adsorbent, K_F_ and n are the Freundlich constants, and C is the concentration of the adsorbed acid dye.

### 2.3. Testing Methods

#### 2.3.1. Fruit Bromelain Activity Assay

Bromelain, present in pineapple juice, belongs to the group of protease enzymes. The analysis of Bromelain activity in fresh pineapple juice was thus conducted by following Sigma’s non-specific protease activity assay [52], using casein as a substrate and *L*-tyrosine as a standard. Casein was hydrolyzed into amino acid tyrosine, which later reacted with Folin phenol to form a blue-colored compound. The absorbance of this compound was measured at a wavelength of 660 nm. The effect of pineapple juice preparation conditions on enzyme activity was assessed. The resulting data were analyzed using a *t*-test statistical method at a 95% confidence interval with SPSS Version 26.

#### 2.3.2. Measurement of Protein Content in Degumming Solutions

The protein content released into water during the degumming process was analyzed by Lowry’s method [53] using the Lowry Reagent and Bovine Serum Albumin (BSA) was employed as a standard. The absorbance of protein solution was monitored at a wavelength of 550 nm.

#### 2.3.3. Surface Morphology Study

The surface characteristics of the silk and adsorbent materials were recorded with Quanta 450 on an FEI scanning electron microscope (SEM) (FEI Company, OR, USA). The analysis of surface area and distribution of the adsorbent particles was monitored from SEM images of the 100 particles at 100× magnification with Image J program, version 1.53.

#### 2.3.4. Water Absorption

The water absorption of silk was assessed according to the AATCC test method 79-2000, Absorbency of Bleached Textiles [54].

#### 2.3.5. Tensile Properties of Silk

The determination of the maximum load and the % elongation of silk filaments was conducted based on ASTM standard D3822-01 (the standard test method for tensile properties of single-textile fibers) [55] using Instron Universal Testing Machine, model 5567, with a 10 N load cell at a tension rate of 20 mm/min. The resulting data were analyzed statistically at a 95% confidence interval with SPSS Version 26.

#### 2.3.6. Sericin Dye Staining of Silk

The direct dye, C.I. Direct Red 80, was used to evaluate the sericin content left on the silk filament. Sericin content is related to the color strength of the dye when used on silk [21]. In this experiment, the silk filaments were stained with 2% of C.I. Direct Red 80 at a liquor ratio of 40:1 at 100 °C for 2 min using an infrared dyeing machine. After that, the silk was rinsed with water and dried at room temperature. The color strength (K/S value) of the stained silk was measured with Datacolor^®^, model 550 spectrophotometer (Datacolor, NJ, USA). The K/S values were calculated as per the Kubelka–Munk equation (Equation (5))

(5) $$K/S=\frac{{\left(1-R\right)}^{2}}{2R}$$

where K is the absorption coefficient, S is the scattering coefficient, and R is the observed reflectance value of the dye fabric.

#### 2.3.7. Surface Area and Porosity Analysis

The analysis of surface area was performed, and the porosity of the adsorbent from pineapple pulp was examined, based on the BET equation—using a gas sorption analyzer, model 3Flex Surface–characterization, from Micromeritics—against those of the tamarind seed testa [46,47] and activated carbon adsorbents.

## 3. Results and Discussion

### 3.1. Bromelain Activity

Bromelain is a group of proteolytic enzymes, and its optimal activity is seen within a temperature range of 40–60 °C and pH range of 3–9 [56]. At present, bromelain from fresh cold-pressed pineapple juice can be extracted using various methods such as centrifugation, ultrafiltration, and lyophilization [57,58], which involve multiple preparation steps. In this study, we analyzed the enzymatic activity of bromelain extracted from fresh pineapple juice. Pineapple juice was isolated by two different methods, viz., filtering through a 60T mesh screen fabric and centrifugation at 4000 rpm for 10 min. The bromelain activity of the filtered juice was 16.40 ± 0.22 µg/mL·min, which showed no significant difference from the value obtained via the centrifugation method (15.76 ± 0.80 µg/mL·min) at a 95% confidence level (*p* = 0.000). However, filtering through the 60 T mesh screen fabric is simpler, faster, and more energy-saving than centrifugation method.

**Figure 3:**
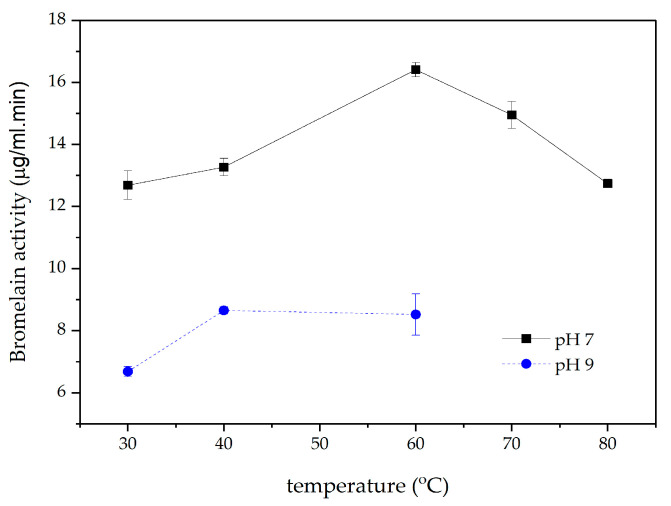
Enzyme activity of bromelain in pineapple juice.

This study also evaluated bromelain activity under pH conditions of 7 and 9 at temperatures ranging from 30 to 80 °C, as depicted in Figure 3. The optimal conditions for bromelain activity in Smooth Cayene pineapple juice were a of pH 7 and a temperature of 60 °C, with the highest enzyme activity of 16.41 µg/mL·min. Ketnawa et al. (2012) reported that the optimal pH for bromelain activity in local pineapples from Chiang Rai province, Thailand, ranged from pH 6.5 to 8.0, with the highest efficiency seen in the vicinity of pH 7.0 [59].

### 3.2. Optimal Degumming Condition with Pineapple Juice

**Figure 4:**
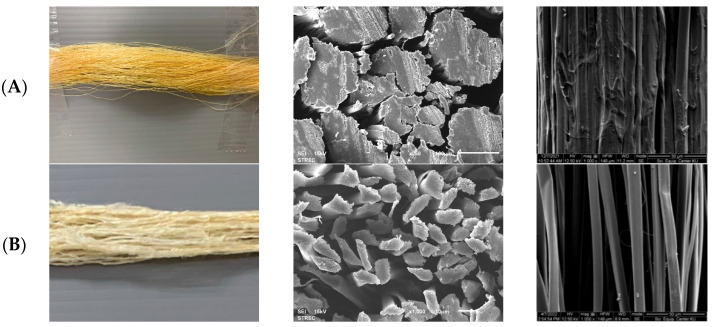
Silk filaments: (**A**) raw silk filaments; (**B**) degummed silk.

The outermost layer of silk filaments is inherently coated with sericin protein [49]. The removal of sericin must be conducted to enhance softness, luster, and the dye/moisture absorption ability to silk. Raw silk typically consists of about 17–38% sericin [49]. This content glues silk fibroin filaments together, as seen in Figure 4. The direct dye staining method, as performed with C.I. Direct Red 80, is commonly used to evaluate the amount of sericin on silk filaments [21]. Raw silk from Chaiyaphum province, Thailand, exhibited dye staining at the color strength (K/S) of 7.41, indicating a large amount of sericin was present on the filaments. When degumming was performed using pineapple juice, sericin was presumably eliminated in the solution, as observed from the drastically reduced dye staining on degummed silk filaments (K/S less than 1). This shows that the fresh pineapple juice [60] can act as a bio-degumming agent with effectively sericin removal from silk filaments.

From the results on the efficiency of bromelain activity in pineapple juice, we found that the optimal conditions were a pH of 7 and a temperature of 60 °C [61]. The effect of degumming times on silk is shown in Table 1. As the duration of degumming process with pineapple juice increased, the degumming efficiency improved. This result corresponds to an increase in water absorption and protein content in the solution, as well as a decrease in the degree of dye staining by sericin present on silk filaments.

**Figure 5:**
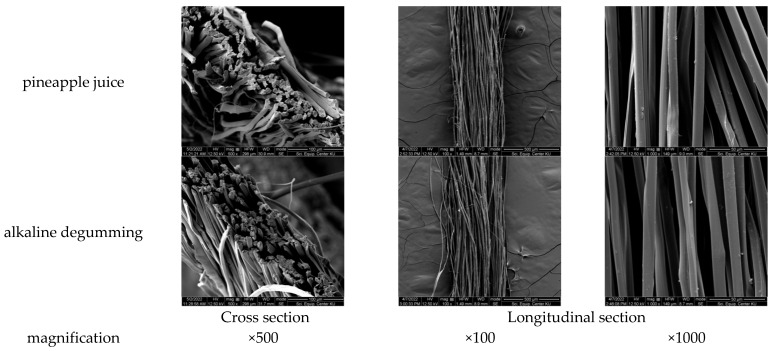
Surface morphology of silk filaments after being pretreated with pineapple juice and alkaline degumming processes.

The results of degumming silk with pineapple juice compared to the typically used alkaline degumming method are shown in Table 2. It was found that alkaline degumming could better eliminate the substances (E) from silk and resulted in a higher protein content in the solution as compared to degumming with pineapple juice. When considering the surface characteristics of the silk filaments after degumming with both methods, the filaments were clean, and virtually no residual sericin was found (Figure 5). However, the silk filaments degummed with alkaline method appeared to be entangled and intermingled, with a significantly lower tensile strength (maximum load) than the silk degummed with pineapple juice at a 95% confidence interval. This may be explained by the breakage of peptide bonds in silk caused by an alkali, leading to more brittle and less shiny filaments with lower strength values than those of their pineapple juice counterparts [62]. Therefore, the enzyme or pineapple juice degumming process is a better alternative for sericin removal without damaging the silk filaments. According to the standard specifications for water absorption (values less than 5 s), it is an effective way of silk preteatment prior to the dyeing process, promoting a minimal long-term environmental impact.

### 3.3. Acid Dye Adsorption Efficiency of Pineapple Pulp

**Figure 6:**
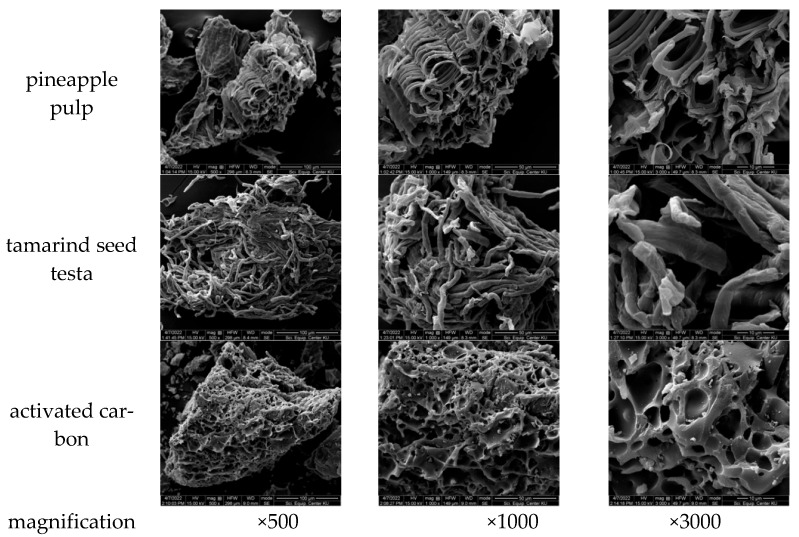
SEM images of the adsorbent materials.

**Figure 7:**
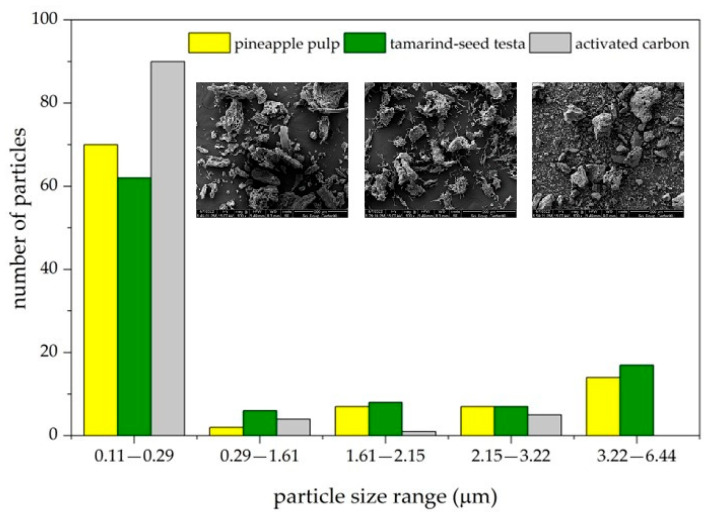
The relationship between the average surface area and the number of particles of the adsorbent materials.

About 15% of dyes cause environmental pollution because they are not easily disposed of and are harmful to aquatic ecosystems, the environment, and nearby communities [40,41]. In addition, certain dye structures or precursors, as well as those from the molecular breakdown of dyes, may lead to the formation of carcinogenic substances. This is harmful to humans, particularly when water-soluble dyes, such as reactive and acid dyes, are produced. The most suitable treatment process for these dyes is adsorption [42], especially the use of adsorbent materials derived from agricultural wastes. Dye molecules are trapped in the adsorbent materials via the adsorption process with agricultural wastes without being converted into harmful substances. This method requires low levels of energy consumption and involves cheap agricultural waste exploitation [44]. Adsorbent materials with high porosity and large surface areas directly influence adsorption efficiency [45]. After peeling, the pineapple by-product was about 39% pulp. After we eliminated sugars, dried it, and ground it finely, it yielded a light-yellow powder of lignocellulosic substances [9,16], comprising 2.53% of the mass by pineapple fruit weight or 6.50% of the mass by pineapple pulp weight. This pineapple pulp powder can be used to adsorb acid dyes in leftover dyeing solutions after silk dyeing. Figure 6 shows the morphological characteristics of the adsorbent material prepared from pineapple pulp, which displays a porous structure, resulting from the orderly arrangement of fibers. The adsorbent from tamarind seed testa [46,47] showed a bundle structure of entangled fibers, and the commercial activated carbon exhibited a relatively porous surface. The results of the average surface area analysis, measured by particle count per 100 adsorbent particles (using Image J Version 1.53), depicted in Figure 7, informed us that the adsorbent particles from both waste materials had an average surface area per particle ranging from 3.22 to 6.44 m^2^, while activated carbon had an average surface area ranging from 0.11 to 0.29 m^2^. The adsorbent material from pineapple pulp had a pore size of 6.1485 nm, a total pore volume of 0.0053 cm^2^/g, and a BET surface area of 3.62 m^2^/g.

**Figure 8:**
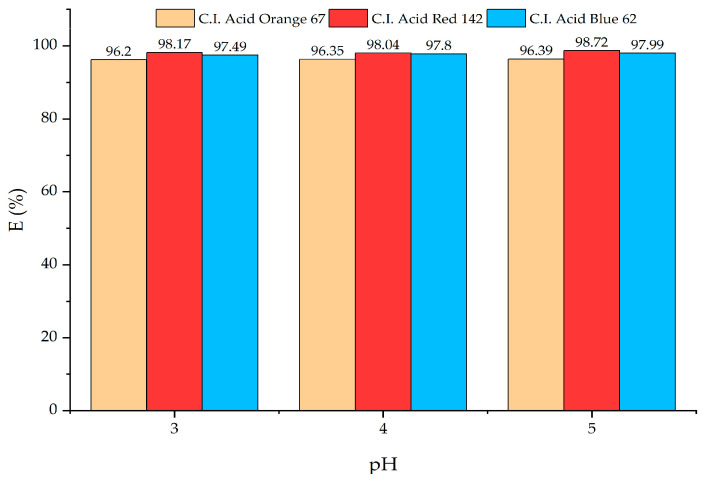
The effect of solution pH on the adsorption efficiency of acid dyes.

The pH values of the dye solution are an important factor, affecting the adsorption efficiency of agricultural adsorbent materials [63], which are primarily composed of lignocellulosic compounds, with negatively charged molecules [64]. Acid dyes have a negatively charged structure. Therefore, the adsorption efficiency of the adsorbent material was expectedly higher in an acidic environment. The adsorbent material derived from pineapple pulp could effectively adsorb all three acid dyes under acidic conditions (pH 3–5), with an adsorption efficiency higher than 95% at room temperature within 24 h, as seen in Figure 8. The optimal pH for acid dye adsorption using the adsorbent prepared from pineapple pulp was pH 5. In such a pH condition, acid dye adsorption would be more simply facilitated as it was within the pH range used for silk dyeing (pH 4–5). Hence, the adsorbent derived from pineapple pulp could be used for acid dye adsorption with no need for dyebath pH adjustment.

**Figure 9:**
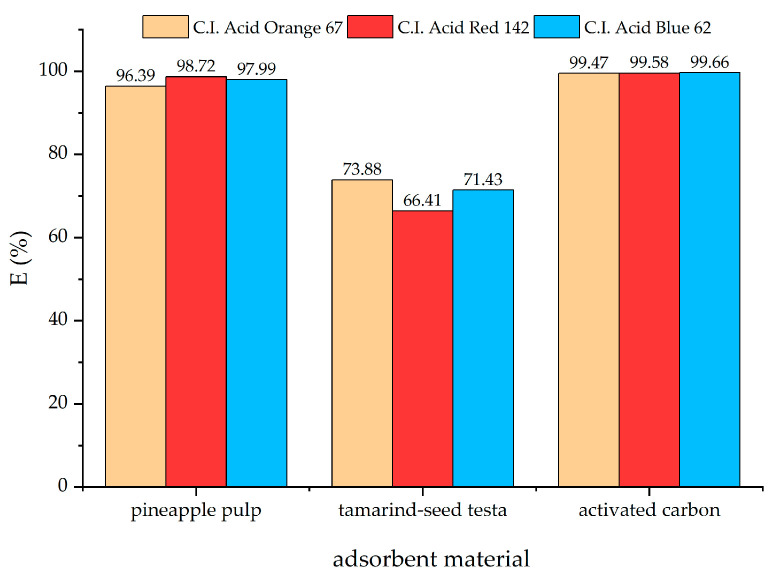
Adsorption efficiency of adsorbent materials prepared from pineapple pulp residue, tamarind seed testa, and activated carbon (pH 5, 24 h).

**Figure 10:**
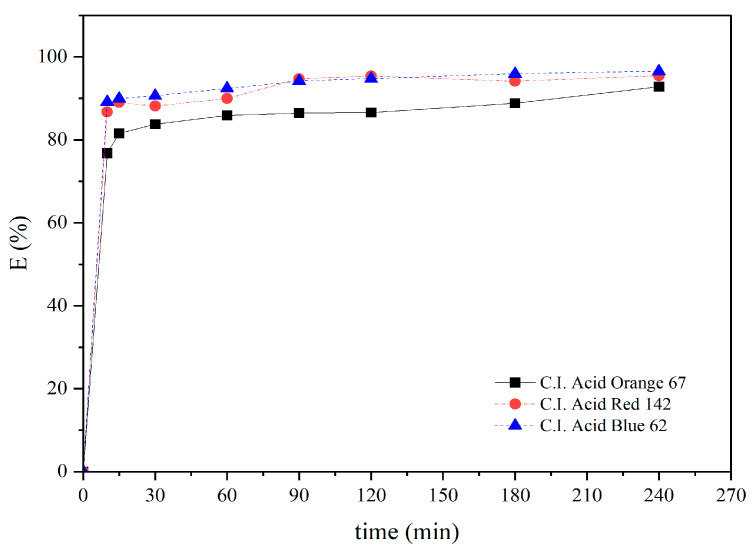
Adsorption efficiency at varying adsorption times.

It was also found that the adsorbent prepared from pineapple pulp showed a comparable adsorption efficiency to that of activated carbon and one higher than that of the tamarind seed testa (Figure 9). The adsorption efficiency of the pineapple pulp adsorbent increased rapidly within the first 15 min, even without any mechanical agitation. During the initial stage of adsorption, there were numerous available adsorption sites on the adsorbent material, resulting in a high adsorption rate within the first 60 min. After that, the adsorption rate gradually decreased and then reached equilibrium within 90 min (Figure 10).

**Figure 11:**
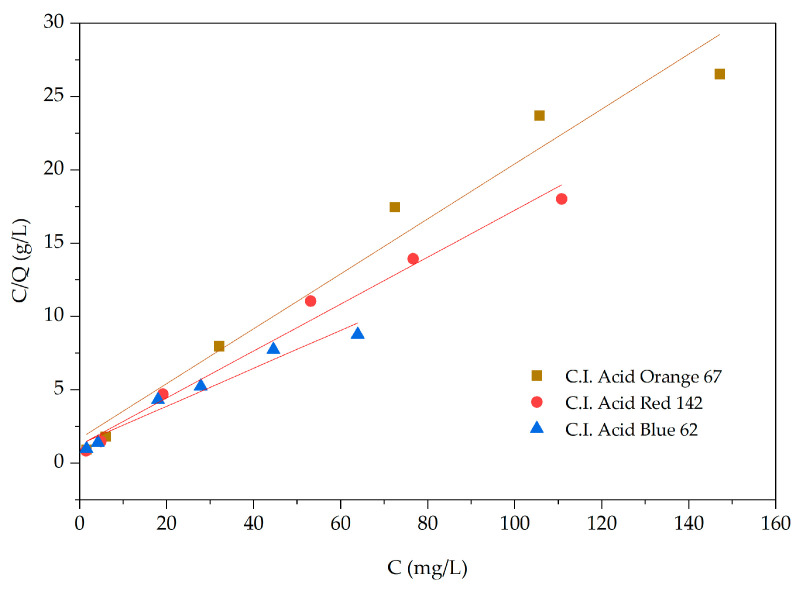
Langmuir adsorption isotherms.

**Figure 12:**
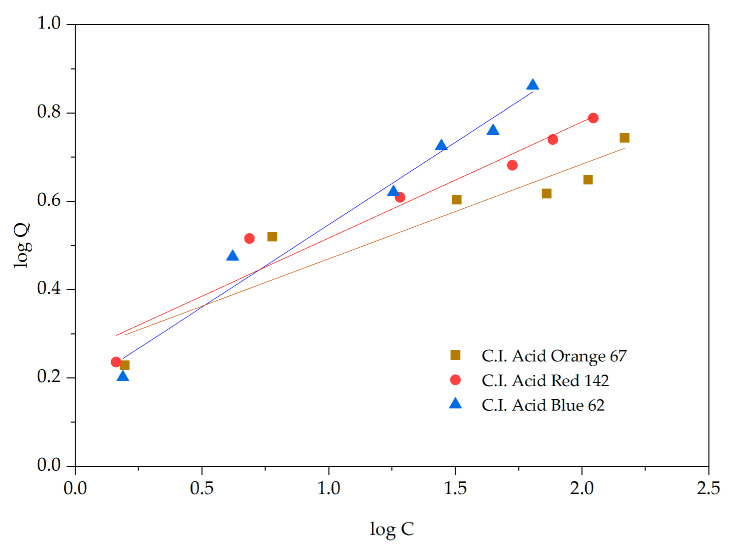
Freundlich adsorption isotherms.

The adsorption behavior of acid dyes by the adsorbent material derived from pineapple pulp was investigated according to Langmuir and Freundlich isotherms using dye solutions at a pH of 5 and an adsorption time of 90 min (equilibrium adsorption) (Figure 11 and Figure 12). We found that C.I. Acid Orange 67 exhibited monolayer adsorption according to the Langmuir isotherm, with a maximum adsorption capacity (q_max_) of 0.60 mg/g of adsorbent material. On the contrary, C.I. Acid Red 142 and C.I. Acid Blue 62 displayed both monolayer and multilayer adsorptions, following the Langmuir and Freundlich isotherms, with maximum adsorption capacities (q_max_) of 0.80 and 0.78 mg/g of adsorbent material, respectively (Table 3).

## 4. Conclusions

The utilization of pineapple in the textile industry primarily focuses on the production of fibers from pineapple leaves. Pineapple fruit is mainly used in the processed food industry. Thailand, a major global exporter of pineapples, recently faced the problem of pineapple oversupply, resulting in price lowering: a large amount of pineapple was left as waste. This issue was addressed, and an alternative way of valorizing pineapple fruit waste was used for greener (silk) textile production. Thai silk fabrics are produced locally by farmer communities, reflecting the cultural identity of each region of Thailand.

In this work, focusing on Thai silk, the utilization of pineapple juice and pulp was studied for use as bio-degumming agents and bio-adsorbent materials for dye adsorption. It was found that using pineapple juice for sericin removal, instead of alkaline degumming, not only employed a natural substance and reduced the amount of residual chemicals released into the environment, but also helped to maintain the quality of the silk, particularly its strength, which directly affected the durability of the silk fabric. However, relatively short shelf life is a major drawback of pineapple juice. Therefore, it would be beneficial to further study enzyme properties and perform the thermodynamic determination of pineapple juice bromelain against various other parameters to gain an insight into the feasibility of prolonging its degumming efficiency.

In addition, pineapple pulp can be used as an adsorbent material from agricultural waste for acid dyeing effluent treatment, as it has a simple adsorption process that can be performed following the silk dyeing process. It was initially found that the adsorbent material had an ability to release dye and reuse. Further studies are needed to attain an understanding of the factors that affect dye release and the cost-effectiveness of reusing adsorbent material or creating new products from cellulose fiber waste. This also includes developing a more concrete method for dye effluent treatment.

## Figures and Tables

**Table 1:** Silk degumming results with pineapple juice at pH 7, 60 °C.

Property	Degumming Time (min)			
	20	40	60	80
Silk filaments	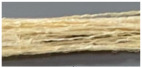	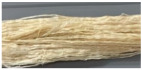	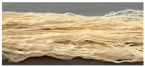	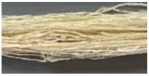
Water absorption (sec)	~5	~5	~2	~2
E (%)	14.53 ± 1.17	19.73 ± 0.20	22.14± 0.60	19.82 ± 0.21
Protein content in solution (ppm)	7964 ± 55	8584 ± 397	9485 ± 304	8861 ± 240
Color strength (K/S)	0.44	0.42	0.30	0.32
Sericin dye staining	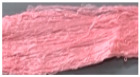	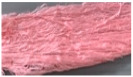	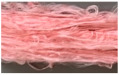	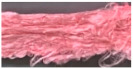
Maximum load (N)	6.53 ± 0.73	5.68 ± 0.51	7.80 ± 1.32	6.5 ± 0.90
Elongation (%)	14.12 ± 2.25	15.68 ± 2.14	20.50 ± 2.00	20.21 ± 5.16

**Table 2:** Properties of silks degummed with pineapple juice as compared to alkaline degumming.

Property	Degumming Conditions	
	Pineapple Juice, pH 7 60 °C, 60 min	2 g/L Na_2_CO_3_, 10 g/L Wetting Agent 95 °C, 45 min
Water absorption (sec)	~2	~2
E (%)	22.14± 0.60	26.35± 0.84
Protein content in solution (ppm)	9485 ± 304	12,718 ± 135
Color strength (K/S)	0.30	0.27
Sericin dye staining	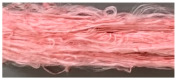	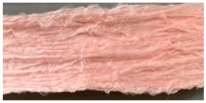
Maximum load (N)	7.80 ± 1.32	6.18 ± 1.56
Elongation (%)	20.50 ± 2.00	20.69 ± 3.83

**Table 3:** Correlation constants of the adsorption models of acid dyes according to Langmuir and Freundlich isotherms.

Acid Dye	Langmuir Isotherms			Freundlich Isotherms		
	q_max_	K_L_	R^2^	n	K_F_	R^2^
C.I. Acid Orange 67	0.60	8.94	0.9686	4.76	1.80	0.8836
C.I. Acid Red 142	0.80	7.78	0.9852	3.85	1.80	0.9437
C.I. Acid blue 62	0.78	9.93	0.9577	2.70	1.49	0.9710

## Data Availability

The original contributions presented in this study are included in the article. Further inquiries can be directed to the corresponding author.

## References

[1] Paz-Arteaga S.L., Cadena-Chamorro E., Goméz-García R., Serna-Cock L., Aguilar C.N., Torres-León C. (2024). Unraveling the Valorization Potential of Pineapple Waste to Obtain Value-Added Products towards a Sustainable Circular Bioeconomy. Sustainability.

[2] Hossain M. (2015). Nutritional Value and Medicinal Benefits of Pineapple. Int. J. Nutr. Food Sci..

[3] Mohd Ali M., Hashim N., Abd Aziz S., Lasekan O. (2020). Pineapple (*Ananas comosus*): A comprehensive review of nutritional values, volatile compounds, health benefits, and potential food products. Food Res. Int..

[4] Moore A., Khanna D. (2023). The Role of Vitamin C in Human Immunity and Its Treatment Potential Against COVID-19: A Review Article. Cureus.

[5] Uckiah A., Goburdhun D., Ruggoo A. (2009). Vitamin C content during processing and storage of pineapple. Nutr. Food Sci..

[6] Aili Hamzah A.F., Hamzah M.H., Che Man H., Jamali N.S., Siajam S.I., Ismail M.H. (2021). Recent Updates on the Conversion of Pineapple Waste (Ananas comosus) to Value-Added Products, Future Perspectives and Challenges. Agronomy.

[7] Beattie J., Quoc T. (2000). Manganese in pineapple juices. Food Chem..

[8] Wang C., Zhu Y., Long H., Ou M., Zhao S. (2022). Relationship between blood manganese and bone mineral density and bone mineral content in adults: A population-based cross-sectional study. PLoS ONE.

[9] Jirapornvaree I., Suppadit T., Popan A. (2017). Use of pineapple waste for production of decomposable pots. Int. J. Recycl. Org. Waste Agric..

[10] Ritthisorn S., Juthakanok R., Teekha C. (2016). Production of pineapple peel handicraft paper from canned fruit industrial factory. Sci. Technol. RMUTT J..

[11] Roda A., Lambri M. (2019). Food uses of pineapple waste and by-products: A review. Int. J. Food Sci. Technol..

[12] Choonut A., Saejong M., Sangkharak K. (2014). The Production of Ethanol and Hydrogen from Pineapple Peel by Saccharomyces Cerevisiae and Enterobacter Aerogenes. Energy Procedia.

[13] Casabar J., Unpaprom Y., Ramaraj R. (2019). Fermentation of pineapple fruit peel wastes for bioethanol production. Waste Biomass Valorization.

[14] Kumar J., Alam I., Kumar A., Kumar A., Singh S.K., Singh S.P., Sharma C. (2024). Sustainable utilization of pineapple fruit waste as a potential source of regenerated cellulose for the development of high-strength paper. Biomass Bioenergy.

[15] Fouda Mbanga B.G., Tywabi-Ngeva Z. (2022). Application of Pineapple Waste to the Removal of Toxic Contaminants: A Review. Toxics.

[16] Sukruansuwan V., Napathorn S.C. (2018). Use of agro-industrial residue from the canned pineapple industry for polyhydroxybutyrate production by Cupriavidus necator strain A-04. Biotechnol. Biofuels.

[17] Rahman M., Bhowmik A., Das S., Chowhan K., Biswas T. (2020). Green Degumming of Silk by Enzyme Extracted from Natural Sources. J. Mater. Sci. Chem. Eng..

[18] Sibaly S., Jeetah P. (2017). Production of paper from pineapple leaves. J. Environ. Chem. Eng..

[19] Jalil M.A., Repon M.R., Jurkonienė S., Haji A., Hussain S.Z., Shukhratov S. (2024). Valorization of pineapple leaves: Effective conversion of agro waste to textile materials. Energy Sci. Eng..

[20] Uzumcu B., Kaplan M., Borazan I. Wild Silk Fibers: Types, Properties and Utilization Areas. Proceedings of the International Congress on Wool and Luxury Fibres.

[21] Kwak H.W., Lee K. (2015). Monitoring of phase separation between silk fibroin and sericin using various dye system. Int. J. Ind. Entomol..

[22] Patichol P., Wongsurawat W., Johri L. (2014). Upgrade strategies in the Thai silk industry: Balancing value promotion and cultural heritage. J. Fash. Mark. Manag..

[23] Wongseelashote B. (2018). Thai silk. J. Silk.

[24] Silva A.S., Costa E.C., Reis S., Spencer C., Calhelha R.C., Miguel S.P., Ribeiro M.P., Barros L., Vaz J.A., Coutinho P. (2022). Silk Sericin: A Promising Sustainable Biomaterial for Biomedical and Pharmaceutical Applications. Polymers.

[25] Gupta D., Agrawal A., Chaudhary H., Gulrajani M., Gupta C. (2013). Cleaner process for extraction of sericin using infrared. J. Clean. Prod..

[26] Lamboni L., Gauthier M., Yang G., Wang Q. (2015). Silk sericin: A versatile material for tissue engineering and drug delivery. Biotechnol. Adv..

[27] Adugna H., Muleta M., Deso L., Hamda A., Adugna A., Edae I., Jifara B., Lemessa R. (2024). Innovative Uses of Agricultural By-Products in the Food and Beverage Sector: A Review. Food Chem. Adv..

[28] Weber C.T., Trierweiler L.F., Trierweiler J.O. (2020). Food waste biorefinery advocating circular economy: Bioethanol and distilled beverage from sweet potato. J. Clean. Prod..

[29] Wedamulla N.E., Fan M., Choi Y.-J., Kim E.-K. (2022). Citrus peel as a renewable bioresource: Transforming waste to food additives. J. Funct. Foods.

[30] Ninpetch U., Tsukada M., Promboon A. (2015). Mechanical Properties of Silk Fabric Degummed with Bromelain. J. Eng. Fibers Fabr..

[31] Freddi G., Mossotti R., Innocenti R. (2003). Degumming of silk fabric with several proteases. J. Biotechnol..

[32] Lo C.-h. (2020). Degumming silk by CO 2 supercritical fluid and their dyeing ability with plant indigo. Int. J. Cloth. Sci. Technol..

[33] Lo C.-H., Chao Y. (2017). Degumming of Silk Fibers by CO_2_ Supercritical Fluid. J. Mater. Sci. Chem. Eng..

[34] Wang R., Zhu Y., Shi Z., Jiang W., Liu X., Ni Q.-Q. (2018). Degumming of raw silk via steam treatment. J. Clean. Prod..

[35] Mahmoodi N.M., Mazaheri F., Rahimi S. (2010). Degradation of sericin (degumming) of Persian silk by ultrasound and enzymes as a cleaner and environmentally friendly process. J. Clean. Prod..

[36] Zhu L., Lin J., Pei L., Luo Y., Li D., Huang Z. (2022). Recent Advances in Environmentally Friendly and Green Degumming Processes of Silk for Textile and Non-Textile Applications. Polymers.

[37] Misran E., Idris A., Mat sarip S.h., Ya’akob H. (2019). Properties of bromelain extract from different parts of the pineapple variety Morris. Biocatal. Agric. Biotechnol..

[38] Bala M., Ismail N.A., Mel M., Jami M.S., Salleh H.M., Amid A. (2012). Bromelain Production: Current Trends and Perspective. Arch. Des Sci..

[39] Ana C., Yeable J.G., Mauricio B., Lidieth U., Marina L., Roos M., Lio Y. (2020). Production of renewable fuel and value-added bioproducts using pineapple leaves in Costa Rica. Biomass Bioenergy.

[40] Noreen S., Tahira M., Ghamkhar M., Hafiz I., Bhatti H.N., Nadeem R., Murtaza M.A., Yaseen M., Sheikh A.A., Naseem Z. (2021). Treatment of textile wastewater containing acid dye using novel polymeric graphene oxide nanocomposites (GO/PAN,GO/PPy, GO/PSty). J. Mater. Res. Technol..

[41] Amalina F., Razak A.S.A., Krishnan S., Zularisam A.W., Nasrullah M. (2022). Dyes removal from textile wastewater by agricultural waste as an absorbent—A review. Clean. Waste Syst..

[42] Bello M.M., Raman A.A.A. (2019). Synergy of adsorption and advanced oxidation processes in recalcitrant wastewater treatment. Environ. Chem. Lett..

[43] Aljeddani G.S., Alghanmi R.M., Hamouda R.A. (2023). Study on the Isotherms, Kinetics, and Thermodynamics of Adsorption of Crystal Violet Dye Using Ag-NPs-Loaded Cellulose Derived from Peanut-Husk Agro-Waste. Polymers.

[44] Abdulhameed A.S., Al Omari R.H., Bourchak M., Abdullah S., Abualhaija M., Algburi S. (2024). Bio-sourced multifunctional adsorbent of chitosan and adipic acid activated-dragon fruit peels for organic dye removal from water: Eco-friendly management and valorization of biomass. Biomass Bioenergy.

[45] Alcalde-Garcia F., Prasher S., Kaliaguine S., Tavares J.R., Dumont M.-J. (2023). Desorption Strategies and Reusability of Biopolymeric Adsorbents and Semisynthetic Derivatives in Hydrogel and Hydrogel Composites Used in Adsorption Processes. ACS Eng. Au.

[46] Chaiyapongputti P., Sae-Bae P., Setthayanond J., Munsuwan P. (2014). Development of adsorbent material from tamarind-seed testa for reactive dye adsorption. Appl. Mech. Mater..

[47] Setthayanond J., Sae-Bae P., Chaiyapongputti P., Lim P. (2017). Chromium (VI) adsorption study using bio-adsorbent material derived from tamarind-seed testa. Key Eng. Mater..

[48] Czaplicki Z., Gliścińska E., Machnowski W. (2021). Natural Silk—An Unusual Fibre: Origin, Processing and World Production. Fibres Text. East. Eur..

[49] Gulrajani M. (2008). Degumming of silk. Rev. Prog. Color. Relat. Top..

[50] Talebpour F., Veysian S.M., Golfazani M.E.H. (2013). Degumming of silk yarn using alkali, enzyme and Seidlitzia rosmarinus. J. Text. Polym..

[51] Wu H., Zhou J., Zhu P., Li J., Li Y. (2024). An Exploration of Alkaline Degumming in the Printing and Dyeing Process of Silk Georgette. Polymers.

[52] Cupp-Enyard C. (2008). Sigma’s Non-Specific Protease Activity Assay—Casein as a Substrate. J. Vis. Exp. JoVE.

[53] Lowry O., Rosebrough N., Farr A.L., Randall R. (1951). Protein measurement with the folin phenol reagent. J. Biol. Chem..

[54] AATCC (2000). Absorbency of Bleached Textiles. AATCC Test Method79-2000.

[55] (2014). Standard Test Method for Tensile Properties of Single Textile Fibers.

[56] Verma V., Singhal G., Joshi S., Choudhary M., Srivastava N., Mir S.A., Manickavasagan A., Shah M.A. (2022). Chapter 10—Plant extracts as enzymes. Plant Extracts: Applications in the Food Industry.

[57] Maurer H. (2001). Bromelain: Biochemistry, pharmacology and medical use. Cell. Mol. Life Sci. CMLS.

[58] Pavan R., Jain S., Shraddha, Kumar A. (2012). Properties and therapeutic application of bromelain: A review. Biotechnol. Res. Int..

[59] Ketnawa S., Chaiwut P., Rawdkuen S. (2012). Pineapple wastes: A potential source for bromelain extraction. Food Bioprod. Process..

[60] Jutamongkon R. (2010). Factors Affecting the Stability of Fruit Bromelain in Smooth Cayenne Pineapple (Ananas comosus). Ph.D. Thesis.

[61] Chuenjai P., Setthayanond J., Tooptompong P. Environmental value creation of degumming process for Thai silk by utilizing pineapple juice. Proceedings of the Pure and Applied Chemistry International Conference 2022.

[62] Khan M.M.R., Tsukada M., Gotoh Y., Morikawa H., Freddi G., Shiozaki H. (2010). Physical properties and dyeability of silk fibers degummed with citric acid. Bioresour. Technol..

[63] Kali A., Amar A., Loulidi I., Hadey C., Jabri M., Alrashdi A.A., Lgaz H., Sadoq M., El-Kordy A., Boukhlifi F. (2022). Efficient Adsorption Removal of an Anionic Azo Dye by Lignocellulosic Waste Material and Sludge Recycling into Combustible Briquettes. Colloids Interfaces.

[64] Bulgariu L., Escudero L., Bello O.S., Iqbal M., Nisar J., Adegoke K.A., Alakhras F., Kornaros M., Anastopoulos L. (2019). The utilization of leaf-based adsorbents for dyes removal: A review. J. Mol. Liq..

